# Supplementary material for: Reproducible and predictable reorganization of place fields driven by grid subfield rate changes
Source: bioRxiv. 2026 Feb 12:2026.02.10.705142. Preprint. [Version 1] doi: 10.64898/2026.02.10.705142 (PMC12918893; doi:10.64898/2026.02.10.705142)
Supplement: Supplement 1 [file NIHPP2026.02.10.705142v1-supplement-1.pdf]

bioRxiv preprint doi: https://doi.org/10.64898/2026.02.10.705142; this version posted February 12, 2026. The copyright holder for this preprint (which was not certified by peer review) is the author/funder, who has granted bioRxiv a license to display the preprint in perpetuity. It is made available under aCC-BY 4.0 International license.

749 # Supplementary Figures

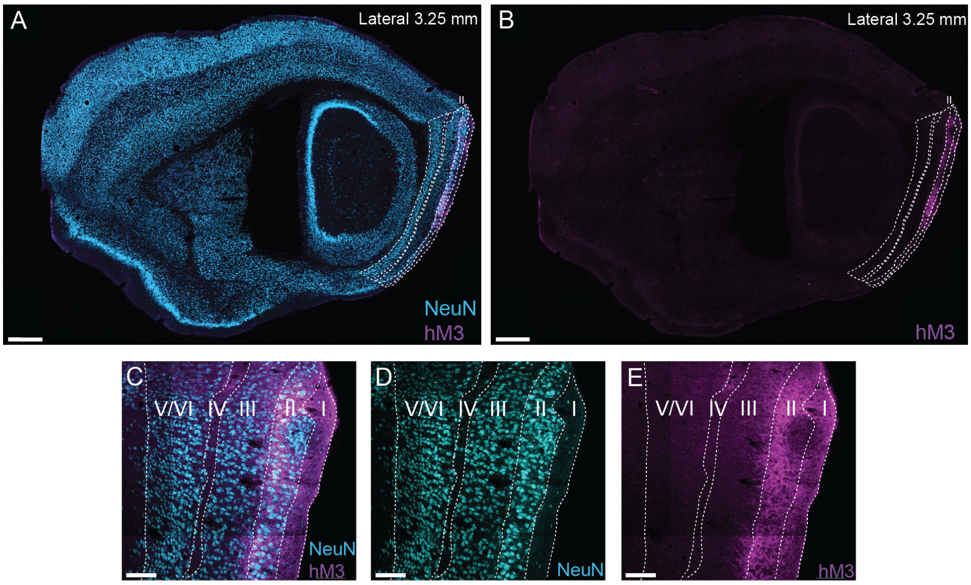

Figure S1. Transgenic expression of hM3Dq DREADD receptor in MEC LII
A) Representative image of fluorescent immunohistochemistry of a sagittal brain slice targeting the transgene hM3Dq (magenta). Expression is largely restricted to entorhinal cortex layer II. Counterstain NeuN (cyan). Scale bar: 500 µm. **B)** Same as in (A) without the NeuN counterstain. **C-E)** Representative high magnification images of dorsal MEC showing NeuN (cyan) and hM3 (magenta) staining. Cropped images from the same brain-wide section shown in (A) and (B). Scale bar: 100 µm.

bioRxiv preprint doi: https://doi.org/10.64898/2026.02.10.705142; this version posted February 12, 2026. The copyright holder for this preprint (which was not certified by peer review) is the author/funder, who has granted bioRxiv a license to display the preprint in perpetuity. It is made available under aCC-BY 4.0 International license.

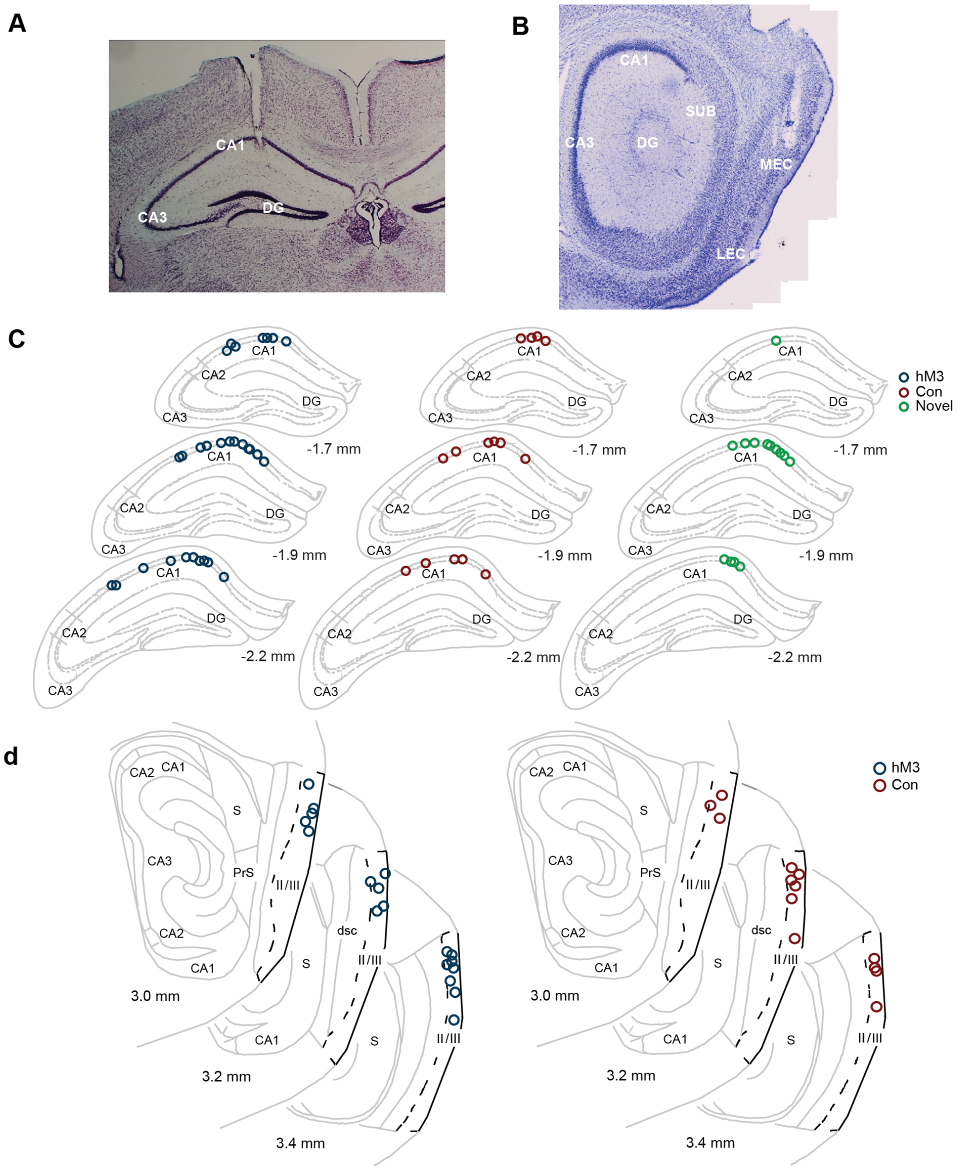

Figure S2. Recording sites in CA1 and MEC.
A) Representative coronal section used to identify tetrode tracks in CA1. **B)** Representative sagittal section used to identify tetrode tracks in superficial MEC. **C)** Tetrode locations in CA1 identified in three coronal sections in hM3 mice (left, blue), control mice (middle, red), and a separate group of mice exposed to two distinct environments (right, green). Numbers indicate distance from bregma. **D)** Tetrode locations in superficial layers (II/III) of MEC identified in three sagittal sections in hM3 (left, blue) and control (right, red) mice. Numbers indicate distance from midline. SUB, subiculum; PrS, presubiculum; dsc, lamina desiccans.

bioRxiv preprint doi: https://doi.org/10.64898/2026.02.10.705142; this version posted February 12, 2026. The copyright holder for this preprint (which was not certified by peer review) is the author/funder, who has granted bioRxiv a license to display the preprint in perpetuity. It is made available under aCC-BY 4.0 International license.

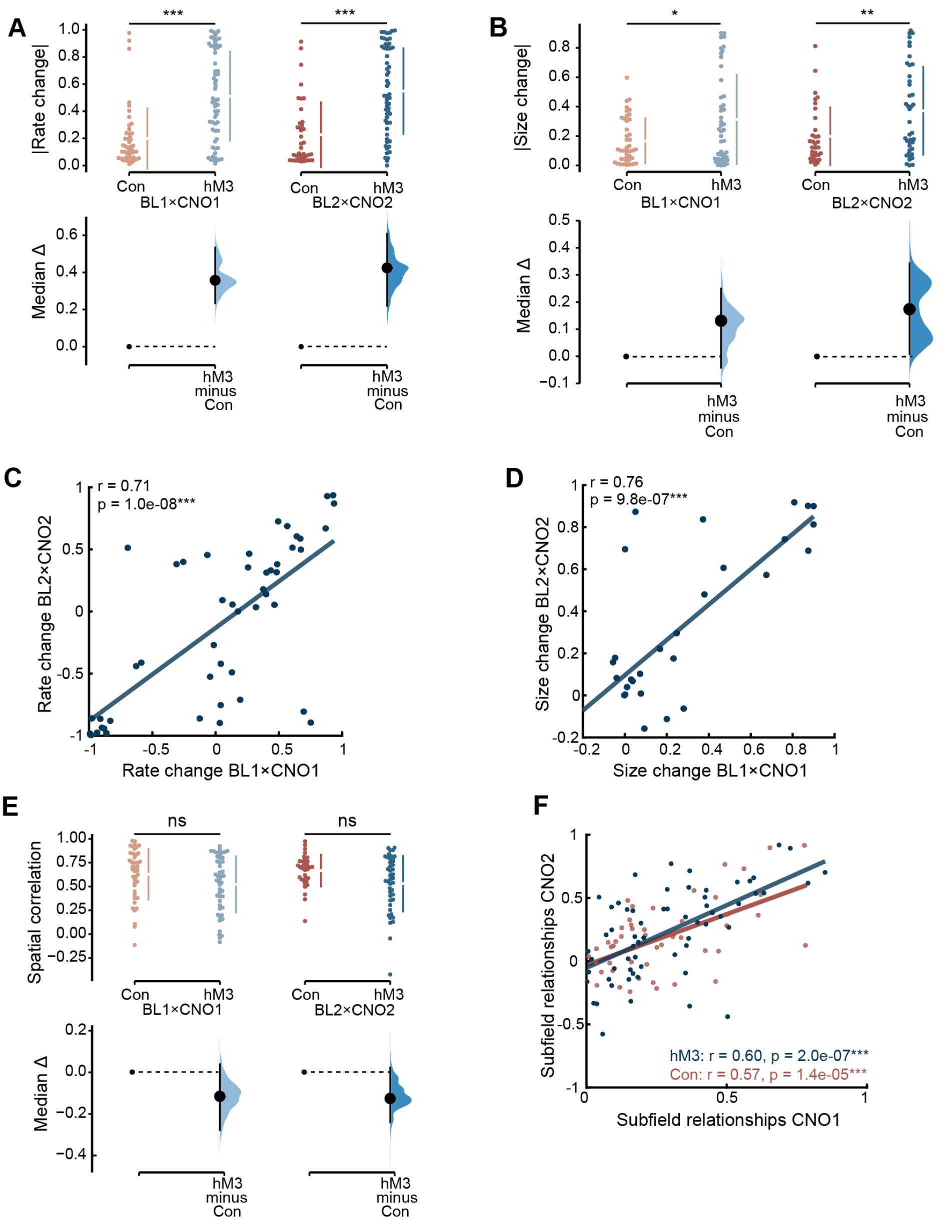

Figure S3. Putative excitatory neurons in MEC exhibit similar changes in firing rate and field size across days.
A-B) Panels show significant changes in firing rate (A, left) and field size (B, right) of putative excitatory neurons in MEC between BL and CNO sessions on Day 1 and Day 2 in hM3 (blue) versus Con (red) mice (rate change, hM3 vs. Con: BL1×CNO1, hM3 n = 61, Con n = 46, Z = 4.8, p = 8.8 x 10^{-7}; BL2×CNO2, hM3 n = 58, Con n = 39, Z = 5.0, p = 3.3 x10^{-7}; size change, hM3 vs. Con: BL1×CNO1, hM3 n = 46, Con n = 42, Z = 1.7, p = 0.04; BL2×CNO2, hM3 n = 41, Con n = 33, Z = 2.5, p = 6.6 x 10^{-3}; one-sided Wilcoxon rank sum tests). Points represent individual MEC neurons; gapped lines represent mean ± standard deviation. Change refers to an absolute difference score (see **Methods**). **C-D)** Scatterplots show significant correlation between firing rate (C, left) and field size (D, right) changes of putative excitatory neurons in MEC between BL and CNO sessions on Day 1 and Day 2 in hM3 mice (rate change, BL1×CNO1 vs. BL2×CNO2: n = 49, r = 0.71, p = 1.0 x 10^{-8}; size change, BL1×CNO1 vs. BL2×CNO2: n = 30, r = 0.76, p = 9.8 x 10^{-7}; linear correlations). Points represent individual MEC neurons. ****p* < 0.001. **E)** Panel shows that there was no difference in the spatial correlation of MEC neurons (including grid cells) following CNO injection between hM3 and control mice (spatial correlation, hM3 vs. Con: BL1×CNO1, hM3 n = 48, Con n = 42, Z = 1.6; p = 0.10; BL2×CNO2, hM3 n = 43, Con n = 34, Z = 1.8; p = 0.07; two-sided Wilcoxon rank sum tests). Points represent individual MEC neurons; gapped lines represent mean ± standard deviation. **F)** Scatterplot showing significant correlation between grid subfield relationships during CNO session on Day 1 and Day 2 in hM3 (blue) and Con mice (red) (CNO1 vs. CNO2: hM3 n = 66, r = 0.60, p = 2.0 x 10^{-7}; Con n = 64, r = 0.57, p = 1.4 x 10^{-5}; linear correlations). For each grid cell, subfield rates were ordered from highest to lowest. We then calculated the normalized difference in peak firing rate between all subfield pairs. Points represent the normalized difference between each subfield pair in CNO1 versus CNO2. ****p* < 0.001. For lower panels in (A), (B) and (E), black dot: median; black bars: 95% confidence interval; filled curve: sampling-error distribution.

bioRxiv preprint doi: https://doi.org/10.64898/2026.02.10.705142; this version posted February 12, 2026. The copyright holder for this preprint (which was not certified by peer review) is the author/funder, who has granted bioRxiv a license to display the preprint in perpetuity. It is made available under aCC-BY 4.0 International license.

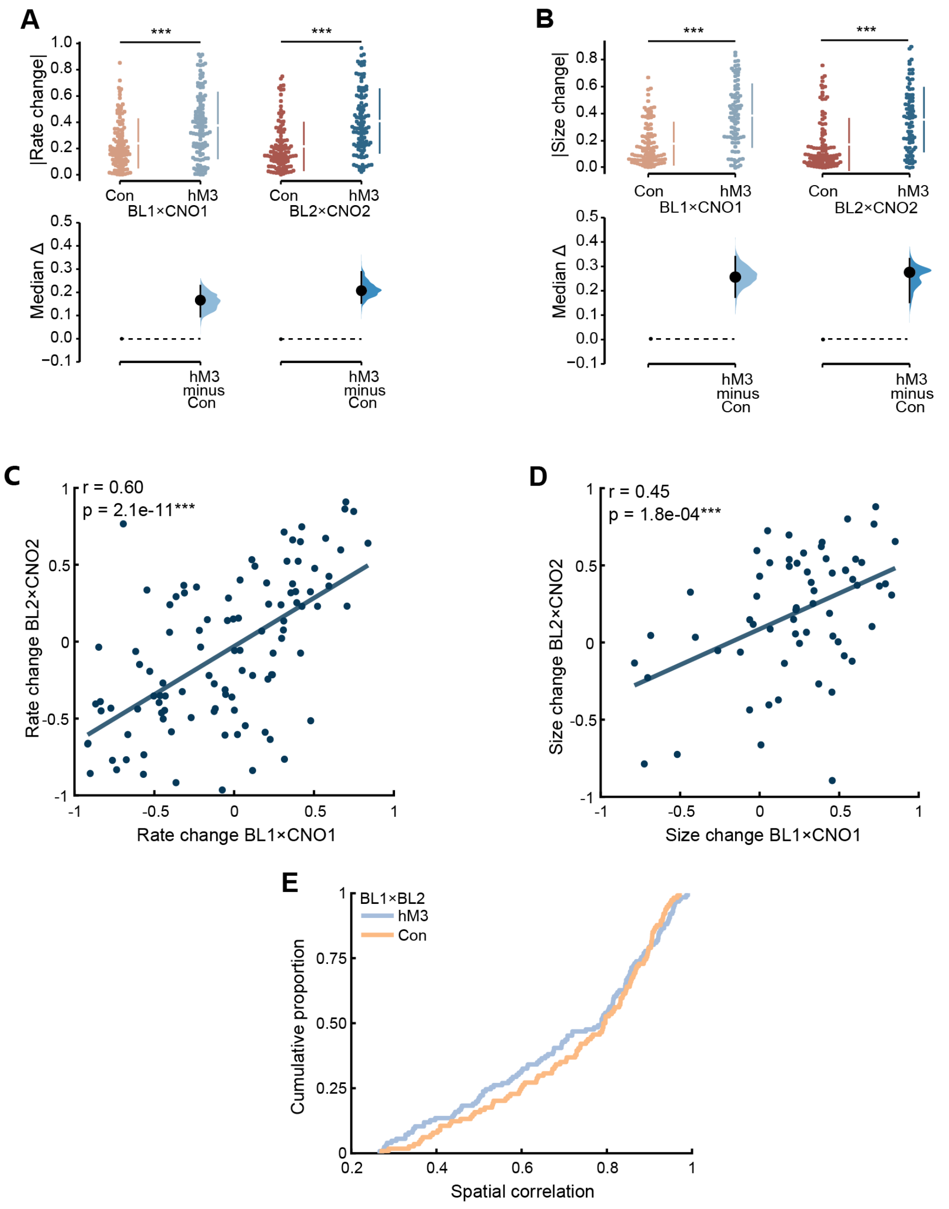

Figure S4. CA1 place cells exhibit consistent changes in firing rate and field size across days.
A-B) Panels show significant changes in firing rate (A, left) and field size (B, right) of CA1 place cells between BL and CNO sessions on Day 1 and Day 2 in hM3 (blue) versus Con (red) mice (rate change, hM3 vs. Con: BL1×CNO1, hM3 n = 113, Con n = 109, Z = 4.1, p = 1.8 x 10^{-5}; BL2×CNO2, hM3 n = 109, Con n = 111, Z = 6.3, p = 1.6 x 10^{-10}; size change, hM3 vs. Con: BL1×CNO1, hM3 n = 86, Con n = 101, Z = 6.1; p = 4.6 x 10^{-10}; BL2×CNO2, hM3 n = 80, Con n = 102, Z = 5.5, p = 2.0 x 10^{-8}; one-sided Wilcoxon rank sum tests). Points represent CA1 place cells; gapped lines represent mean ± standard deviation. Change refers to an absolute difference score (see **Methods**). **C-D)** Scatterplots show significant correlation between firing rate (C, left) and field size (D, right) changes of CA1 place cells between BL and CNO sessions on Day 1 and Day 2 in hM3 mice (rate change, BL1×CNO1 vs. BL2×CNO2: n = 104, r = 0.60, p = 2.1 x 10^{-11}; size change, BL×CNO1 vs. BL×CNO2: n = 64, r = 0.45, p = 1.8 x 10^{-4}; linear correlations). Points represent CA1 place cells. **E)** Cumulative distribution function shows spatial correlation of rate maps from BL sessions on each recording day for place cells in hM3 (light blue) and Con (light orange) mice. Note that there was no difference between groups (spatial correlation, BL1×BL2: hM3 n = 126, median = 0.79, 95% CI, 0.69 – 0.82; Con n = 114, median = 0.79, 95% CI, 0.74 – 0.83; hM3 vs. Con, D* = 0.10, p = 0.57, two-sided Kolmogorov-Smirnov test), indicating that place cells in hM3 mice returned to their BL representations 12+ hrs. after CNO injection. For lower panels in (A) and (B), black dot: median; black bars: 95% confidence interval; filled curve: sampling-error distribution.

bioRxiv preprint doi: https://doi.org/10.64898/2026.02.10.705142; this version posted February 12, 2026. The copyright holder for this preprint (which was not certified by peer review) is the author/funder, who has granted bioRxiv a license to display the preprint in perpetuity. It is made available under aCC-BY 4.0 International license.

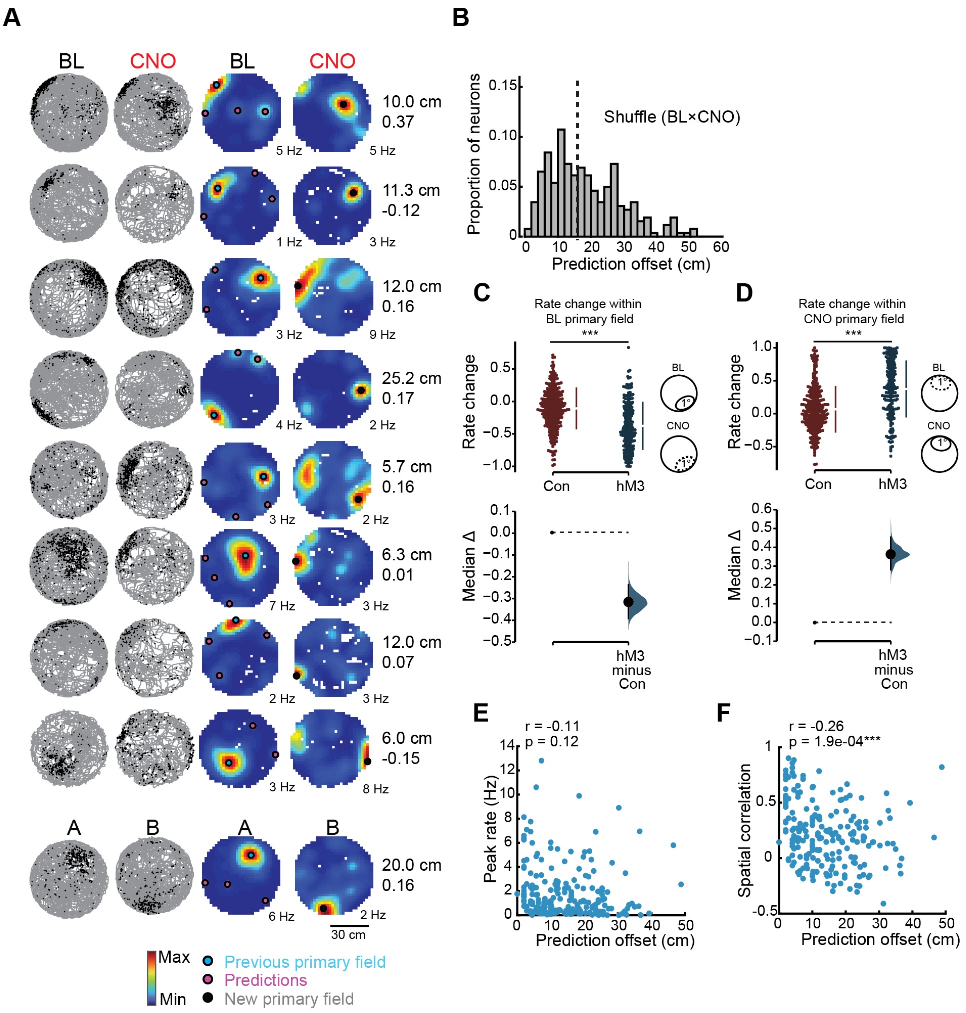

Figure S5. Predictable reorganization of place code despite robust artificial remapping.
A) First eight rows show spike path plots and firing rate maps of BL and CNO sessions for eight CA1 place cells in hM3 mice (one cell per row). In spike path plots (first two columns), the mouse's trajectory is shown in gray and action potentials are represented in black. For firing rate maps (last two columns), color indicates firing rate. Peak firing rate is noted below each rate map. Same convention for place field prediction as in Figure 3A (blue circles, primary place field in BL session; pink circles, predictions of place field location; black circles, primary place field in CNO session). The prediction offset and the spatial correlation between rate maps from the BL and CNO sessions are shown on the right for each cell. Bottom row shows spike path plots and firing rate maps for one CA1 place cell from a control mouse exposed to two distinct environments (A and B). Same convention as above. **B)** Histogram shows prediction offsets for a shuffled control dataset (see **Methods**). Note that prediction offsets were significantly lower in hM3 mice (see Figure 3C) than in the shuffled dataset (hM3 n = 204, median = 12.1 cm, 95% CI, 10.2 – 14.6 cm; shuffle n = 261, median = 16.1 cm, 95% CI, 14.4 – 17.9 cm; hM3 vs. shuffle, Z = 3.9, p = 5.6 x 10^{-5}, one-sided Wilcoxon rank sum test). Vertical lines represent the median of the distribution. **C)** Panel shows the firing rate change within the primary place field from the BL session for place cells in hM3 (blue) and Con (red) mice. Between sessions, there was a significant decrease in firing rate within the BL primary field in hM3 mice relative to controls (Con n = 320, median = -0.10; hM3 n = 204, median = -0.42; Con vs. hM3, Z = 8.4, p = 5.3 × 10^{-17}, two-sided Wilcoxon rank sum test). Points represent CA1 place cells; gapped lines represent mean ± standard deviation. Change refers to a difference score (see **Methods**). **D)** Panel shows the firing rate change within the primary place field from the CNO session for place cells in hM3 (blue) and Con (red) mice. Between sessions, there was a significant increase in firing rate within the CNO primary field in hM3 mice relative to controls (Con n = 320, median = 0.03; hM3 n = 204, median = 0.39; Con vs. hM3, Z = 8.4, p = 4.25 × 10^{-17}, two-sided Wilcoxon rank sum test). Points represent CA1 place cells; gapped lines represent mean ± standard deviation. Change refers to a difference score (see **Methods**). **E)** Scatterplot shows no relationship between the peak firing rate in the predicted location in the BL session and the prediction offset for place cells in hM3 mice (n = 204, r = -0.11, p = 0.12, linear correlation). Points represent CA1 place cells. **F)** Scatterplot shows weak relationship between the degree of remapping following CNO injection and the prediction offset for place cells in hM3 mice (n = 204, r = -0.26, p = 1.9 × 10^{-4}, linear correlation). Points represent CA1 place cells. ***p < 0.001. For lower panels in (C) and (D), black dot: median; black bars: 95% confidence interval; filled curve: sampling-error distribution.

bioRxiv preprint doi: https://doi.org/10.64898/2026.02.10.705142; this version posted February 12, 2026. The copyright holder for this preprint (which was not certified by peer review) is the author/funder, who has granted bioRxiv a license to display the preprint in perpetuity. It is made available under aCC-BY 4.0 International license.

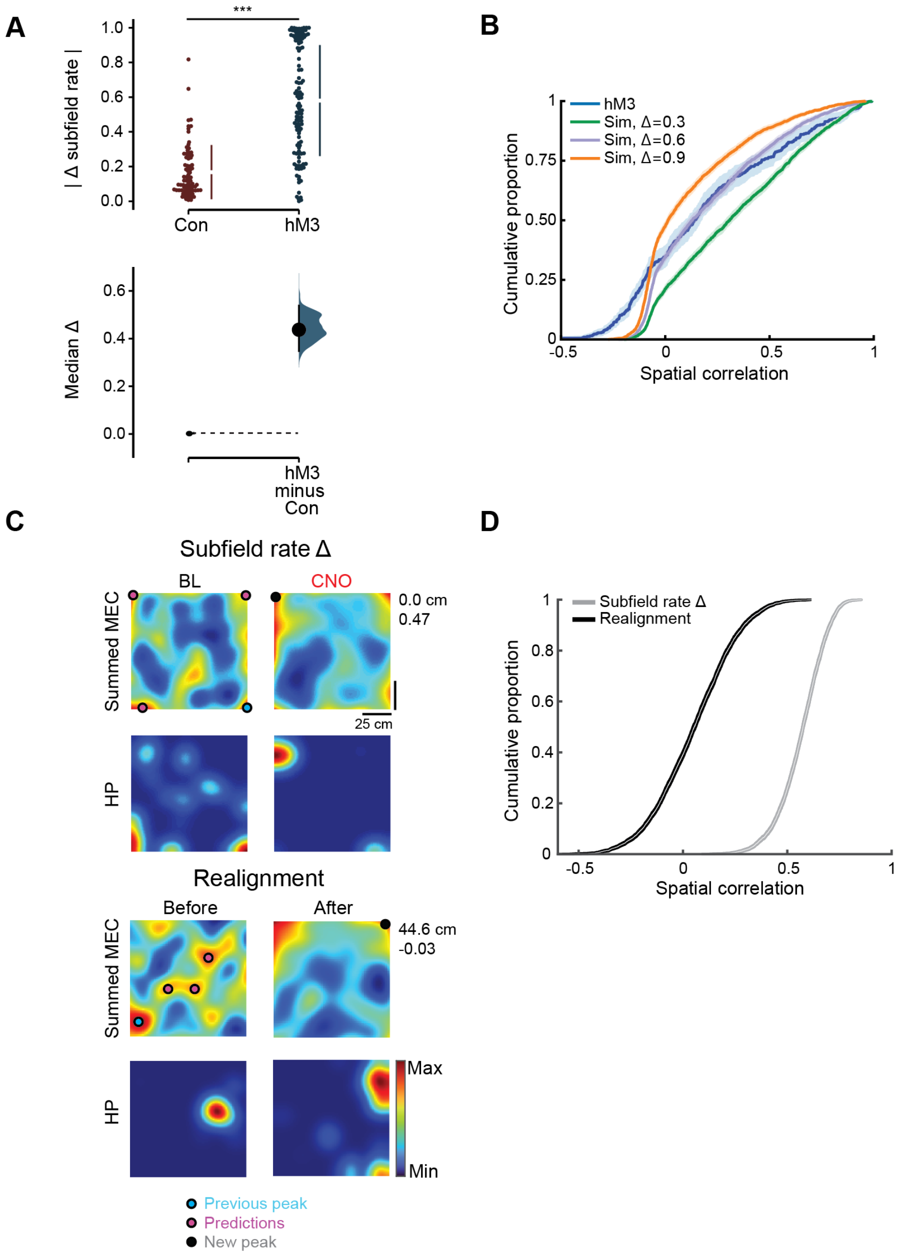

Figure S6. Grid subfield rate changes redistribute activity among spatially stable grid inputs.

A) Panel shows distribution of grid subfield rate changes in hM3 (blue) and Con (red) mice between BL and CNO sessions. In our simulation of the grid-to-place cell transformation, grid subfield rates were modified by values drawn randomly from the distribution of grid field rate changes in hM3 mice (median = 0.55, 95% CI, 0.48 – 0.65). Note that there was significantly more change in grid subfield rates between BL and CNO sessions in hM3 mice relative to controls (hM3 vs. Con: BL×CNO, hM3 n = 117, Con = 80, $p = 2.7 \times 10^{-19}$, two-sided Wilcoxon rank sum test). Top, points represent grid subfields; gapped lines represent mean ± standard deviation. Change refers to an absolute difference score (see **Methods**). Bottom, test statistic is the median difference, shown on the y axis as a bootstrap sampling distribution. Black dot: median; black bars: 95% confidence interval; filled curve: sampling-error distribution. p value is from Wilcoxon rank sum test; ***$p < 0.001$. **B)**Cumulative distribution function shows spatial correlation between rate maps from BL and CNO sessions for place cells in hM3 mice (blue) and simulated place cells. When grid subfield rates were modified by values drawn randomly from the distribution of grid field rate changes in hM3 mice (Sim Δ = 0.6, purple), the extent of remapping was similar for simulated place cells and place cells in hM3 mice (spatial correlation, BL×CNO: hM3 n = 394, median = 0.14, 95% CI, 0.09 – 0.17; Sim Δ = 0.6 n = 1,975, median = 0.13, 95% CI, 0.11 – 0.16; hM3 vs. Sim Δ = 0.6, Z = 1.7, p = 0.09, two-sided Wilcoxon rank sum test). Decreasing (Sim Δ = 0.3, green) or increasing (Sim Δ = 0.9, orange) the extent of grid subfield rate changes (by adjusting the median of the distribution from which subfield rates were sampled) modulated the degree of remapping among simulated place cells (spatial correlation, BL×CNO: Sim Δ = 0.9 n = 2,033, median = 0.01, 95% CI, 0.00 – 0.03; Sim Δ = 0.3 n = 1,897, median = 0.31, 95% CI, 0.28 – 0.33). **C)** Panels show excitation maps representing the summed grid cell input to a single simulated place cell before and after grid subfield rate change (top) or independent realignment of grid modules (bottom). Excitation maps (top row) depict strength of summed grid input (from blue to red). Color in corresponding place cell rate maps (bottom row) indicates firing rate. To predict changes in the location of summed grid inputs (rather than hippocampal place fields), we used the same method as in Figure 3A (blue circles, previous peak; pink circles, predictions; black circles, new peak). The prediction offset and the spatial correlation between excitation maps from each session are shown on the right. Note that grid subfield rate changes typically caused the location of the primary field to shift to an alternate peak in the input pattern rather than a random location, resulting in low prediction offsets and high spatial correlations between sessions (median prediction offset = 16.0 cm, 95% CI, 13.0 – 19.0 cm). The location of the primary field typically shifted to an unpredicted location following independent realignment of grid modules, resulting in high prediction offsets and low spatial correlations between sessions (median prediction offset = 40.2 cm, 95% CI, 39.0 – 41.2 cm). The prediction offset was significantly lower after grid subfield rate changes than independent realignment (subfield rate change n = 3,730, independent realignment n = 4,616, Z = 21.8, $p = 9.3 \times 10^{-106}$, two-sided Wilcoxon rank sum test). **D)** Cumulative distribution functions show spatial correlation between excitation maps before and after grid subfield rate change (gray) or independent realignment of grid modules (black). Grid subfield rate changes resulted in a predictable reorganization of grid cell input, resulting in significantly higher spatial correlation between excitation maps from each session than following independent realignment (spatial correlation: subfield rate change n = 5,000, median = 0.575, 95% CI, 0.571 – 0.579; independent realignment n = 5,000, median = 0.054, 95% CI, 0.047 – 0.061; subfield rate change vs. independent realignment, D* = 0.91, $p < 2.2 \times 10^{-16}$, two-sided Kolmogorov-Smirnov test).

bioRxiv preprint doi: https://doi.org/10.64898/2026.02.10.705142; this version posted February 12, 2026. The copyright holder for this preprint (which was not certified by peer review) is the author/funder, who has granted bioRxiv a license to display the preprint in perpetuity. It is made available under aCC-BY 4.0 International license.

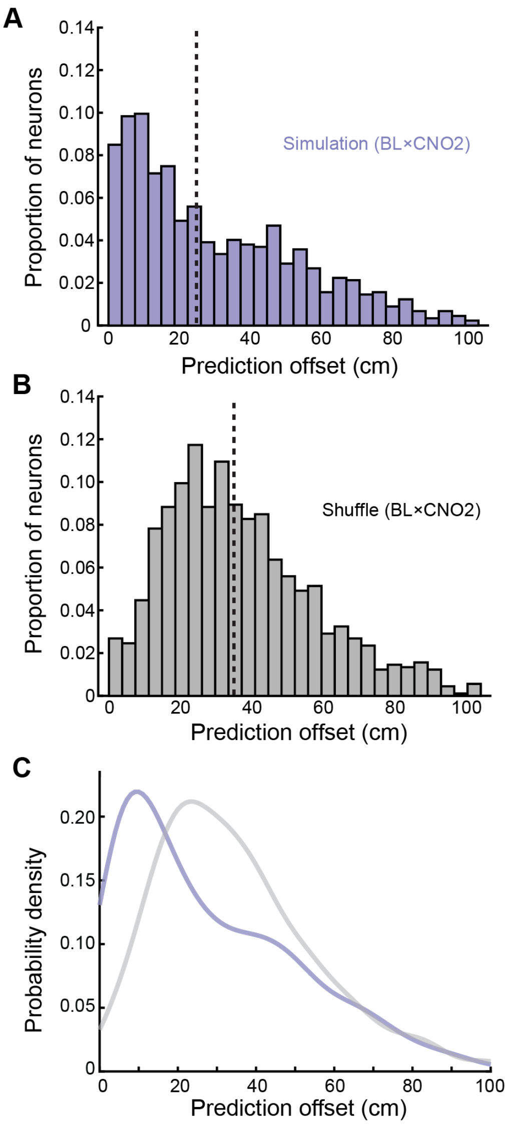

Figure S7. Grid subfield rate changes elicit predictable and reproducible reorganization of hippocampal place fields.
A) Histogram shows prediction offsets between BL and CNO2 for simulated place cells. Note that we were able to predict place field locations equally well during both runs of the simulation (prediction offset: BL×CNO2 n = 895, median = 22.2 cm, 95% CI, 19.4 – 24.1 cm; BL×CNO1 vs. BL×CNO2, Z = 1.0, p = 0.31, two-sided Wilcoxon rank sum test). Vertical line represents the median of the distribution. **B)** Histograms show prediction offsets between BL and CNO2 for a shuffled control dataset (see **Methods**; BL×CNO2 n = 1,218, median = 31.7 cm, 95% CI, 30.2 – 33.1 cm). Note that prediction offsets for simulated place cells between BL and CNO2 were significantly lower for the shuffled control dataset (simulation vs. shuffle, Z = 8.7, p = 4.4 x 10^{-18}, two-sided Wilcoxon rank sum test). Vertical line represents the median of the distribution. **C)** Kernel smoothed density estimate of prediction offset for simulated place cells (purple) and a shuffled control dataset (gray) between the BL session and CNO2 (BL×CNO2).
